# Implementation of a rural emergency department-initiated buprenorphine program in the mountain west: a study protocol

**DOI:** 10.1186/s13722-024-00496-0

**Published:** 2024-09-03

**Authors:** Natasha Seliski, Troy Madsen, Savannah Eley, Jennifer Colosimo, Travis Engar, Adam Gordon, Christinna Barnett, Grace Humiston, Taylor Morsillo, Laura Stolebarger, Marcela C Smid, Gerald Cochran

**Affiliations:** 1 Department of Family and Preventive Medicine, University of Utah School of Medicine, 375 Chipeta Way Suite A, Salt Lake City, UT 84108 USA; 2 Department of Emergency Medicine, University of Utah School of Medicine, 50 N. Medical Drive, Salt Lake City, UT 84132 USA; 3 Wound Care and Hyperbaric Medicine, St. Mark’s Hospital, 1200 East 3900 South #G175, Salt Lake City, UT 8412 USA; 4 Castleview Hospital, 300 N Hospital Drive, Price, UT 84501 USA; 5 Clinical Care, Knowledge, and Advocacy, Department of Internal Medicine, Program for Addiction Research, University of Utah School of Medicine, 30 North Mario Capecchi Drive, 3rd Floor North, Salt Lake City, UT 84112 USA; 6 Informatics, Decision-Enhancement, and Analytic Sciences (IDEAS) Center, VA Salt Lake City Health Care System, 295 Chipeta Way, Salt Lake City, UT 84108 USA; 7 Utah Department of Health and Human Services, 195 North, West, Salt Lake City, UT 1950, 84116 USA; 8 Department of Obstetrics and Gynecology, Division of Maternal Fetal Medicine, School of Medicine, University of Utah Health, 30 North 1900 East #2B200, Salt Lake City, UT 84132 USA

**Keywords:** Buprenorphine, Emergency department, Rural, Opioid use disorder, Implementation

## Abstract

**Background:**

Opioid related overdose morbidity and mortality continue to significantly impact rural communities. Nationwide, emergency departments (EDs) have seen an increase in opioid use disorder (OUD)-related visits compared to other substance use disorders (SUD). ED-initiated buprenorphine is associated with increased treatment engagement at 30 days. However, few studies assess rural ED-initiated buprenorphine implementation, which has unique implementation barriers. This protocol outlines the rationale and methods of a rural ED-initiated buprenorphine program implementation study.

**Methods:**

This is a two-year longitudinal implementation design with repeated qualitative and quantitative measures of an ED-initiated buprenorphine program in the rural Mountain West. The Reach, Effectiveness, Adoption, Implementation, and Maintenance (RE-AIM) framework outlines intervention assessments. The primary outcome is implementation measured by ED-initiated buprenorphine protocol core components. Reach, adoption, and maintenance are secondary outcomes. External facilitators from an academic institution with addiction medicine and prior program implementation expertise partnered with community hospital internal facilitators to form an implementation team. External facilitators provide ongoing support, recommendations, education, and academic detailing. The implementation team designed and implemented the rural ED-initiated buprenorphine program. The program includes OUD screening, low-threshold buprenorphine initiation, naloxone distribution and administration training, and patient navigator incorporation to provide warm hand off referrals for outpatient OUD management. To address rural based implementation barriers, we organized implementation strategies based on Expert Recommendations for Implementing Change (ERIC). Implementation strategies include ED workflow redesign, local needs assessments, ED staff education, hospital leadership and clinical champion involvement, as well as patient and community resources engagement.

**Discussion:**

Most ED-initiated buprenorphine implementation studies have been conducted in urban settings, with few involving rural areas and none have been done in the rural Mountain West. Rural EDs face unique barriers, but tailored implementation strategies with external facilitation support may help address these. This protocol could help identify effective rural ED-initiated buprenorphine implementation strategies to integrate more accessible OUD treatment within rural communities to prevent further morbidity and mortality.

**Trial Registration:**

ClinicalTrials.gov National Clinical Trials, NCT06087991. Registered 11 October 2023 – Retrospectively registered, https://clinicaltrials.gov/study/NCT06087991.

## Introduction

The opioid epidemic remains a significant public health concern as opioid-related overdose deaths have continued to rise in the United States between 1999 and 2021 [1]. Since the onset of the COVID-19 pandemic, emergency departments (EDs) have seen an increase in opioid use disorder (OUD)-related visits compared to other substance use disorders (SUD) [2]. Individuals with non-fatal overdose ED visits related to OUD are at increased risk of subsequent fatal overdose [3]. Therefore, the ED is a critical and opportune setting for OUD interventions.

EDs can serve as an important low barrier means of accessing medication for OUD (MOUD) and resource access. ED-initiated buprenorphine with a brief intervention increases treatment engagement 30 days from discharge compared to outpatient referral alone or facilitated referral with a brief intervention [4]. Additionally, naloxone distribution has been successfully integrated into ED settings [5]. ED-embedded staff members, including case management and peer recovery services, can also assist in providing support and coordinate outpatient interventions including referrals for continued MOUD follow-up [6–8]. Various ED-initiated buprenorphine models have been incorporated into EDs across the United States [9, 10]. However, few studies have assessed rural ED-initiated buprenorphine implementation despite the striking need for services in these communities.

From 1999 to 2019, the overdose mortality rate increased nearly five-fold (4.0 to 19.6 per 100,000) in rural counties compared to over three-fold (6.4 to 22.0 per 100,000) in urban counties [11]. Opioid overdose mortality due to natural and semisynthetic opioids are approximately 13% higher in rural compared to urban regions [12]. While individuals with OUD are also more likely to experience an overdose in rural compared to urban locations, they are less likely to receive naloxone for overdose reversal [13, 14]. OUD related morbidity in rural populations also entails increased risk of Human Immunodeficiency Virus (HIV) and Hepatitis C [15, 16]. Rural residents with OUD seeking treatment face additional barriers including prolonged clinic travel time, transportation difficulties, stigma, and limited MOUD clinician availability [17–19]. These geographical characteristics highlight the need for accessible rural OUD interventions, including ED-initiated buprenorphine, that could decrease associated morbidity and mortality.

Though initial rural ED-initiated buprenorphine studies show promise, [20, 21] implementation has primarily been conducted within urban settings, particularly academic settings [9, 22, 23] However, academic urban centers often have readily available resources including medication access, outpatient follow-up, trained staff, and leadership support [4, 9]. In contrast, rural facilities with fewer resources and support have additional implementation barriers [24]. Rural clinicians likely may also have limited experience with MOUD and providing mental health services [17, 25, 26]. Even with the recent elimination of the Drug Addiction Treatment Act (DATA) 2000 waiver, rural clinicians have consistently indicated the need for more MOUD training [27, 28]. Though buprenorphine can be initiated from an outpatient setting, there are few readily available rural clinicians to provide MOUD follow-up [17, 25, 29]. In addition to training and provider resources, buprenorphine may also be limited within rural regions due to restricted pharmacy availability [30–32]. Further rural ED-initiated buprenorphine implementation methods are needed to address these challenges. This protocol outlines the rationale and methods for an implementation study that incorporates ED-initiated buprenorphine in a rural Mountain West setting.

## Methods

### Design

This is a protocol for a longitudinal implementation design with repeated quantitative and qualitative measures. Over a two-year timeframe, this study aims to implement a rural ED-initiated buprenorphine program and assess outcomes using Reach, Effectiveness, Adoption, Implementation, and Maintenance (RE-AIM) (Table 1) [33, 34]. The primary outcome is implementation measured by the number of patients receiving core services components. This includes the number of patients diagnosed with OUD, discharged with a buprenorphine prescription, given naloxone and overdose reversal training, and follow-up with an MOUD clinician to ensure core components of the ED-initiated buprenorphine protocol are followed. Reach, adoption, and maintenance are secondary outcomes. RE-AIM was selected as it is an efficient framework to plan and assess interventions across diverse clinical and community settings, including a prior successful statewide ED-initiated buprenorphine study [21, 35]. 

We used facilitation to create, adapt, and implement a site-specific ED-initiated buprenorphine program. A prior ED-initiated buprenorphine implementation study successfully used facilitation in low resource high needs settings including one rural facility [24]. Facilitation is where one makes things easier for others [36]. This includes specific facilitator roles as well as facilitation actions to drive successful implementation [37]. Internal and external facilitators formed an implementation team to facilitate the ED-initiated buprenorphine program. This entailed adaptation of an urban academic center ED-initiated buprenorphine protocol to address the core components. Internal facilitators included rural hospital ED nurses, an ED physician, a program coordinator, a patient support navigator, the hospital chief executive officer (CEO), and an outpatient MOUD clinician. External facilitators from the academic institution included an experienced SUD nurse case manager, program coordinators, an experienced researcher with prior implementation experience, and addiction board certified physicians in outpatient internal medicine, emergency medicine, and Maternal Fetal Medicine (MFM). This study and protocol (version 1, updated 4/12/23) was approved by the University of Utah Institutional Review Board.

**Table 1:** The Reach, effectiveness, adoption, implementation, and Maintenance/Sustainability (RE-AIM) framework assessing outcomes of a rural ED-initiated buprenorphine program in the Mountain West [33].

RE-AIM Framework	
Dimension	**Outcomes**
Reach	• Divide the number of patients who received ED-initiated buprenorphine by the total number who were eligible to receive it.
Efficacy	• ED-initiated buprenorphine already has established efficacy demonstrated by D’Onofrio et al.(4)
Adoption	• Annual qualitative semi-structured interviews assessing ED-initiated buprenorphine protocol uptake involving ED staff, leadership, and outpatient community partners. • The total number of ED staff trained in the ED-initiated buprenorphine protocol.
Implementation	• The number of patients diagnosed with OUD, discharged with a buprenorphine prescription and naloxone, trained in naloxone administration, and follow-up with an MOUD clinician to ensure core components of the ED-initiated buprenorphine protocol are followed. • Creation of a clinical workflow by implementation team. • Patient completion of the Patient Satisfaction Questionnaire Short Form (PSQ-18) after discharge. • Patient completion of the Center for Substance Abuse Treatment (CSAT) Government Performance and Results Act (GPRA) Client Outcome Measures for Discretionary Programs upon ED discharge and at 6-months post ED discharge.
Maintenance/ Sustainability	• Ensure a long-term sustainability plan is in place and secure ongoing funding.

ED = Emergency Department, MOUD = Medication for opioid use disorder

### Study setting

An academic medical center approached opinion leaders, including ED staff and administrators, from a rural community hospital to collaborate in forming an implementation team and creating a site-specific ED-initiated buprenorphine program. We selected this rural hospital as it did not have an ED-initiated buprenorphine protocol and has high rates of drug overdose deaths within the two surrounding counties (41.8 and 37.4 deaths per 100,000 people from 2017 to 2021) [38]. It is also the only location in these two counties with an ED. The ED includes 13 beds staffed by one EM physician and two to four nurses. Among the EM physicians, 3 are full-time and 9 are locum tenens. The 2023 ED census was 11,443.

The rural hospital did not have access to addiction medicine, psychiatry specialists, or offer ED-initiated buprenorphine prior to partnership with the academic institution. This program aligns well with rural administrator priorities as ED-initiated buprenorphine has demonstrated to be cost-effective and ED directors have expressed a need to act in combating the opioid epidemic and maintain best practices [39, 40]. Furthermore, ED-clinician barriers to buprenorphine initiation include lack of appropriate training and development of local protocols have been identified as a facilitator [41]. 

Though ED-initiated buprenorphine implementation has been integrated across various geographic locations, this has not been assessed within the rural Mountain West. This region encompasses a large land area with significant opioid overdose mortality [42]. The academic institution and rural community hospital partnered and received funding through the Utah Department of Health and Human Services for associated program initiation costs.

### Participants

#### ED staff

Rural hospital ED staff are involved in implementation and received buprenorphine, protocol, and stigma education. Involved ED staff includes nurses, clinicians, pharmacists, emergency medical technicians, nursing assistants, and health unit coordinators.

#### Patients

Any patient presenting to the rural community ED with opioid-related concerns can initiate buprenorphine, including those with opioid withdrawal, overdose, injection site infections, or other concerns identified throughout the ED encounter. Buprenorphine initiation inclusion criteria includes individuals with moderate to severe OUD per the Diagnostic and Statistical Manual of Mental Disorders Fifth Edition (DSM-5) and agreeable to MOUD treatment with buprenorphine [43]. Exclusion criteria include patients with a history of buprenorphine hypersensitivity. A maternal fetal medicine (MFM) physician who is board certified in addiction medicine provided input prior to implementation and recommended inclusion of pregnant patients. Participants initiated on buprenorphine and further clinical considerations are ultimately at the discretion of the ED clinician.

### Buprenorphine initiation

#### ED-initiated buprenorphine

**Fig. 1:**
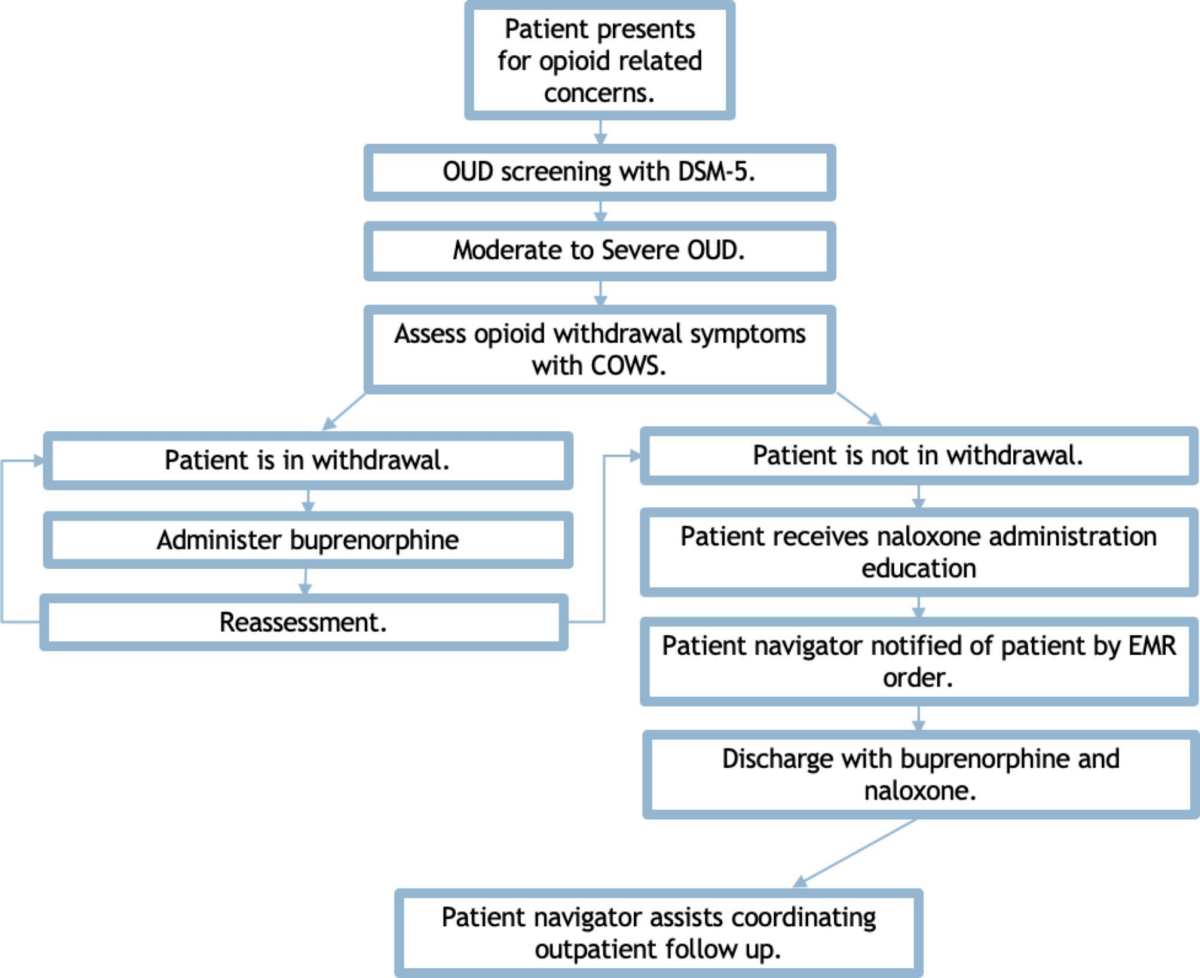
Rural Mountain West ED-initiated buprenorphine workflow overview. DSM-5 = Diagnostic and Statistical Manual of Mental Disorders, 5th Edition, OUD = Opioid Use Disorder, COWS = Clinical Opiate Withdrawal Scale, EMR = Electronic Medical Record

Based on community hospital staff input during pre-implementation weekly meetings, the ED-initiated buprenorphine protocol was modeled after the partnering academic institution ED program that had been successfully implemented. The ED-initiated buprenorphine protocol contains core components from the Yale Clinical Protocol with previously demonstrated efficacy by D’Onofrio et al. [4]. and the American College of Emergency Physicians (ACEP) [44] recommendations that have been adapted to a rural ED community hospital setting. Core model components include OUD screening, distribution and administration training, and warm hand off referral to continued outpatient MOUD (Fig. 1) [45]. 

Any patient presenting to the ED with opioid related concern identified throughout the encounter is screened by ED nursing staff with an OUD DSM-5 checklist [43]. Urine drug screening is not used to determine buprenorphine initiation eligibility to prevent additional barriers. Unfortunately, all ED patients cannot be screened for potential OUD given limited staff and resources at this facility. However, further universal screening could be considered in other facilities. The diagnosis of moderate to severe OUD is then confirmed by an ED physician. ED buprenorphine initiation is ultimately at the discretion of the physician.

Buprenorphine initiation was not limited by the DATA 2000 waiver as this was discontinued shortly before implementation, allowing all rural hospital ED physicians with a Drug Enforcement Agency (DEA) license to prescribe buprenorphine [27]. Due to concerns of limited funding and resources to cover associated costs for individuals presenting with OUD, the hospital decided patients may receive ED-initiated buprenorphine once every six months. However, return to use is a common and expected aspect of SUD management [29]. Patients presenting for opioid-related concerns prior to six months from their initial ED buprenorphine initiation may be restarted based on ED clinician opinion. Other facilities may consider different approaches based on comfort level and resources.

Based on the patient Clinical Opiate Withdrawal Scale (COWS) score calculated by nursing or ED physicians, patients are either initiated on buprenorphine in the ED or receive home buprenorphine initiation instructions [46]. Patients then receive naloxone and overdose reversal training education prior to discharge from nursing, physicians, pharmacists, or the patient navigator. Patients receive a sufficient buprenorphine supply until their initial outpatient MOUD appointment.

Patient navigator referrals are entered by an ED physician through the electronic medical record (EMR). The patient navigator is employed within the same healthcare system as the ED and can access the EMR for the patient’s contact information. The patient navigator visits the patient during their ED visit or contact patients within 24 to 72 h after they are discharged to schedule an outpatient warm handoff referral for ongoing MOUD.

The patient navigator also provides ongoing psychosocial support, outpatient community behavioral health resources, resources for other comorbidities, and Medicaid enrollment assistance for uninsured patients to aid in their recovery. Due to limited staff availability and funding, the patient navigator is only available for variable weekday daytime hours. There is no ED embedded social workers or case managers that are otherwise commonly staffed in urban EDs. The patient is also provided with the patient navigator’s contact information and instructed to contact them for scheduling outpatient follow-up and resources. This is provided in case the patient does not have continuous phone access.

### Implementation strategies

The primary implementation strategy involves expert external facilitators from an academic institution partnering with rural hospital internal facilitators to implement a rural ED-initiated buprenorphine protocol. External facilitators provide ongoing support, recommendations, education, and academic detailing for two years. Academic detailing entails in-person one-on-one education visits providing unbiased evidence-based recommendations between expert external facilitators and rural hospital staff [47]. 

The implementation team will meet on a regular basis throughout the implementation process for a two-year timeframe, to refine the program based on internal and external facilitator feedback. This includes communicating any protocol change updates. Implementation team meetings occur on a weekly basis. Monthly meetings are also attended by clinical experts, with all partnering academic institution and rural community hospital stakeholders, to provide ongoing program feedback and input. Previous successful ED-initiated buprenorphine implementation studies also utilized external facilitators with prior implementation experience and addiction medicine expertise [48–51]. To address implementation barriers, we organized implementation strategies using facilitation based on Expert Recommendations for Implementing Change (ERIC) as it is based on expert consensus in varying contexts (Table 2) [52]. 

**Table 2:** Rural Mountain West ED-initiated buprenorphine barriers and associated implementation strategies organized based on Expert recommendations for Implementing Change (ERIC) [52].

Constructs	Implementation Barriers	Implementation Strategies
Rural ED-Initiated Buprenorphine Protocol	• Insufficient resources, knowledge, or ability to create and implement an ED-initiated buprenorphine protocol.	*Assess and redesign workflow* • An ED workflow with MOUD core components was incorporated. This includes DSM-5 OUD screening, using a COWS assessment with corresponding appropriate buprenorphine dosing, naloxone distribution and training, and a discharge process including a warm handoff referral to an outpatient MOUD clinician. *Tailor strategies* • These include having an EMR ED-initiated buprenorphine order set and ED embedded patient navigator to support the warm handoff referral.
ED Staff	• OUD related stigma, unfamiliarity with protocol, and limited buprenorphine knowledge.	*Develop and distribute education materials* • Educational materials outlining the protocol were posted in the ED, emailed, and uploaded onto the hospital educational portal. *Conduct educational outreach visits* • Further education on the protocol, buprenorphine, and stigma is provided by academic external facilitators including physicians and clinicians through in-person and remote education sessions, staff meeting updates, and academic detailing.
*Community Members and Patient*	• Patients and community members are unaware of ED-initiated buprenorphine program or are not identified as having OUD.	*Involve patients and community members* • Local outreach to outpatient MOUD clinicians, treatment facilities, and social media/newspaper advertisement can be used to spread program awareness. *Redesign workflow* • Patients presenting with opioid related concerns are screened for OUD with the DSM-5.
*Rural Hospital Facility*	• Limited support from ED and hospital leadership, limited hospital medication supply, and culture.	*Involve executive boards and clinical champions* • ED and hospital staff were identified as clinical champions in joining the implementation team to create cultural change. There was hospital and leadership support to encourage ongoing implementation. *Conduct local needs assessment* • An appropriate supply of hospital buprenorphine was confirmed prior to implementation.
*Community Resources*	• Limited outpatient pharmacy medication supply and few MOUD clinicians to provide ongoing MOUD.	*Conduct local needs assessment* • An appropriate supply of community pharmacy buprenorphine was confirmed prior to implementation. *Engage community resources* • Internal facilitators engaged with community partners to establish a partnership with an outpatient MOUD clinician.

COWS = Clinical Opioid Withdrawal Scale, ED = Emergency Department, MOUD = Medication for opioid use disorder, OUD = Opioid Use Disorder

#### ED-initiated buprenorphine protocol

Various implementation strategies address rural-ED initiated buprenorphine protocol barriers within a rural ED facility. Core components from D’Onofrio et al. and ACEP recommendations including OUD screening, a DSM-5 OUD checklist, buprenorphine initiation, naloxone distribution and administration training, and warm hand off outpatient MOUD referral were included in the protocol [4, 43, 44]. 

Clinician unfamiliarity with buprenorphine and the perception that administration is time-consuming have previously been identified as implementation barriers [53]. This is especially pertinent in rural facilities with limited staff availability and resources. A possible facilitator based on previous work includes medical record order sets [20]. Order sets may limit clinician burden in placing referrals to ensure outpatient follow-up given the challenges that exist for rural referral processes [54]. 

The protocol was tailored to the rural ED setting based on pre-implementation weekly implementation team meetings. This included nursing or physician opioid withdrawal scale (COWS) assessment with appropriate corresponding buprenorphine dosing as well as a discharge process with a warm hand off referral to a partnering community MOUD clinician using an ED embedded patient navigator process and EMR order set [46]. EMR order sets can serve as an important tool to facilitate the process of rural ED-initiated buprenorphine given limited staff availability and staffing with locum tenens who may be unfamiliar with the process.

Additional barriers consistent with prior studies include facility SUD resources. and poor referral pathways in rural areas [55, 56]. The rural community hospital has access to one social worker and the ED utilizes an external facility for crisis interventions. In comparison, the partnering urban academic center has 24-hour access to multiple on-site crisis resources including social workers.

ED-integrated patient navigators have been successfully utilized in prior ED-initiated buprenorphine studies [20, 57]. One qualitative study comparing urban to rural ED-based OUD peer service implementation found peer services further adapted to limited rural outpatient services beyond the ED including peer recovery coaching and could relieve ED staff workload [54]. The patient navigator can be vital in providing expedited outpatient care coordination, accessing community recovery resources, establishing patient trust, and supporting patients based on individual needs. This includes providing appropriate patient follow up and SUD resources based on geographic location and financial considerations.

#### ED Staff

To prepare ED staff for ED-initiated buprenorphine program implementation, specific implementation strategies can be used. ED clinicians in previous studies reported limited buprenorphine readiness and lack of knowledge as barriers to ED-initiated buprenorphine [40, 41]. This emphasizes the importance of buprenorphine training and protocol education, especially within limited resource areas. Rural facilities also often see fewer ED patients initiated on buprenorphine compared to urban sites, rural staff and clinicians may not gain familiarity with the process as quickly [24, 54]. Prior research has shown the benefit of academic detailing for ED-initiated buprenorphine implementation, [48, 50] This project heavily relies on clinical experts as external facilitators to provide targeted ED staff education.

We designed and administered staff education sessions for program adoption. The first component of training involved a program collaborator, who is a board-certified addiction medicine specialist from the partnering academic institution, led an introduction to buprenorphine education session. This occurred prior to program implementation for all rural community hospital staff and community members to attend in person or virtually. The ED-embedded patient navigator completed an 8-week online Patient Navigator course and 16 h of Motivational Interview training prior to program implementation.

We also created education materials for distribution based on pre-implementation hospital staff input. These included a buprenorphine and ED workflow outline to distribute to all ED staff including nurses, clinicians, pharmacists, emergency medical technicians, nursing assistants, and health unit coordinators. Materials were distributed through email, posted throughout ED work areas, and presented at a monthly ED nursing staff meeting. The ED nursing director approved and conducted knowledge comprehension sign-off sheets for each ED staff member who was not a physician.

Physicians received a pre-recorded lecture led by an emergency medicine physician from the partnering academic institution who is a clinical expert in ED-initiated buprenorphine. The lecture contains ED clinical workflow integration, buprenorphine mechanism of action, dosing, precautions, and precipitated withdrawal management. The pre-recorded lecture was reviewed by the rural community ED physicians. All physicians and ED staff completed training prior to implementation. We will conduct additional staff training sessions every six months during an ED staff monthly meeting and associated training materials will be emailed to all ED staff. All ED staff are invited to attend with remote or in-person options. Training will also be a part of new staff onboarding.

The external facilitators also provide academic detailing through mentored support to ensure community staff comfort with OUD management. This involves an experienced nurse case manager providing support to the patient navigator. Clinical experts, include an outpatient internal medicine, emergency medicine, and MFM physician who are board certified in addiction medicine provide support to ED and outpatient MOUD clinicians. Education materials and mentored support including web-based discussion and on-demand curated materials offer more training flexibility for rural ED staff where in-person training is geographically limited.

Studies show stigma is often experienced among individual with OUD in rural communities [19, 40, 58–60]. Internal facilitators and community partners identified lack of trust and stigma as barriers to program utilization among patients with OUD. Internal facilitators recommended staff anti-stigma training to attract participants through positive community awareness [21]. In response to this request, the external facilitators requested an anti-stigma training session through state partners for ED staff to build awareness in addressing individuals with SUD and decrease stigma.

#### Community members and patients

Prior studies show limited community program awareness as a barrier in rural settings [40]. Program marketing has been key in ED-buprenorphine implementation studies to encourage engagement [21, 51]. In rural areas with limited OUD treatment access, this can create program awareness among community facilities and individuals with OUD requiring immediate intervention. Our program addressed this barrier and used a local company to integrate familiar community healthcare staff to gain community trust to advertise the ED-initiated buprenorphine program. To create effective program awareness, internal facilitator feedback was used. Among the priority platforms for outreach, the implementation team identified social media advertisement, which is used ubiquitously in the community. This included a short video advertisement with local internal facilitators involved in the program. Additional recommendations included flyer distribution at local facilities, billboards, and local newspaper publications.

Internal facilitators also recommended direct community outreach. Internal facilitators, including hospital staff coordinators, are more likely to be familiar with local facilities and establish connections. To increase program awareness, hospital staff coordinators distributed flyers and discussed the program with community facilities, including local law enforcement agencies, outpatient medical clinics, public health centers, and outpatient chemical dependency treatment facilities and organizations. These methods have potential to increase community and patient trust and awareness.

Eligible ED patients can be further identified through screening. All patients presenting to the ED at any time with opioid related concerns are screened by ED nursing staff with an OUD DSM-5 checklist [43]. This ensures that patients presenting with OUD can be offered ED-initiated buprenorphine with patient navigator support.

#### Rural hospital facility

Barriers within the rural hospital facility needed to be addressed for program implementation. Limited hospital leadership support and mentorship can serve as barriers to ED-initiated buprenorphine, especially in rural areas where few individuals may have a significant impact [40, 41]. The inner context was addressed by having ED and hospital leadership serve as clinical champions to create cultural change and acceptance. ED physicians and nurses, the chief nursing officer (CNO), and CEO serve as key implementation clinical champions in creating a culture of change.

An appropriate hospital pharmacy buprenorphine supply was also confirmed prior to implementation to address internal contexts. Limited buprenorphine supplies occur due to federal and varying state regulations. As buprenorphine is a Schedule III controlled substance, buprenorphine is closely monitored by the United States DEA according to the Controlled Substances Act (CSA) [61]. Per the CSA, DEA licenses have the potential to be revoked among pharmacies that are suspected of diversion if suppliers report a large or suspicious buprenorphine supply ordered [62]. This can result in limited pharmacy buprenorphine dispensing to comply with regulations.

To ensure patients have timely buprenorphine access, we discussed the medication supply with the community hospital pharmacist prior to implementation. The pharmacist confirmed an adequate supply in a partnering outpatient pharmacy, but buprenorphine was not stocked within the hospital pharmacy. Subsequently, the community hospital pharmacist orders and maintains a sufficient buprenorphine supply.

#### Community resources

Limited rural community resources were addressed through partnership with an outpatient MOUD clinician, ensuring adequate financial support, and appropriate community buprenorphine supply. Despite ACEP’s recommendation of having a specified outpatient MOUD referral for patients receiving ED-initiated buprenorphine, [44] outpatient accommodation for patients and referral coordination continue to be ED-initiated buprenorphine implementation barriers [53, 55, 63]. 

We encountered barriers consistent with these findings prior to implementation, including no available partnering community outpatient MOUD clinicians for expedited appointments. In our experience, few local clinicians prescribe buprenorphine and there is limited availability among local outpatient clinicians who did prescribe buprenorphine. We discussed this barrier with the community hospital CEO who was supportive of hiring an outpatient clinician within the same healthcare system to ensure program viability. An advanced practice clinician (APC) was identified as a partnering outpatient MOUD clinician. The APC has dedicated MOUD clinic time once weekly so that follow-up appointments are coordinated within six days of ED discharge.

MOUD access can be cost prohibitive within rural areas facing increasing economic decline with fewer employment opportunities [64]. Dedicated financial resources are needed through grant funding or other sources to support patient costs and hospital resources [41]. This program is funded with a Utah Department of Health and Human Services grant over two years. This provides funding for rural community hospital coordinators, the patient navigator, clinical champions, and academic institution collaborators. The grant funding also covers ED clinician, facility fees, buprenorphine prescriptions, and naloxone kits at no cost to uninsured and underinsured patients. However, a sustainability plan is needed to sustain these components beyond initial grant funding.

Sustainability plans may be helpful for ED-initiated buprenorphine program longevity in areas with few accessible OUD resources supplemented by limited grant funding [17, 25, 26]. This is important as linkage to care, costs, and social determinants of health have been identified as barriers to ED-initiated buprenorphine programs [55]. Given limited time for project support, we created a sustainability plan following the framework set by Shediac-Rizkallah et al., [65] that includes negotiation, effectiveness, financing, and training. Negotiation is incorporated to ensure long-term equal partnership among the external and internal facilitators. This will be accomplished through ongoing meetings to facilitate communication and decision-making involving the implementation team and key community stakeholders. Negotiation is also utilized to ensure community needs are guiding the program. An advisory council was created and consists of community members to provide input so that these needs are met.

Project implementation outcomes are shared with the advisory council during quarterly meetings. These outcomes may demonstrate program benefits to garner potential ongoing financial support through state, county, or national grants. Potential source funding would be identified through a joint effort among advisory council members. External facilitators will further support these endeavors by providing program outcomes and grant application guidance.

Finally, rural community pharmacies may also have limited buprenorphine supplies to comply with state and federal regulations, as previously mentioned [61]. We confirmed buprenorphine availability at community pharmacies prior to program initiation.

### Measured outcomes

The primary study outcome includes rural ED-initiated buprenorphine protocol implementation. This will be measured by the number of patients diagnosed with OUD, discharged with a buprenorphine prescription, naloxone kit, trained in naloxone administration and follow-up with an MOUD clinician (Table 3). We will also use RE-AIM to measure secondary outcomes (Table 1). Reach includes the number of patients who received ED-initiated buprenorphine divided by the total number who were eligible to receive it. Efficacy has been established in prior studies and ED-initiated buprenorphine is the recommended standard of care. [4, 44] We will assess adoption using annual qualitative semi-structured interviews based on Consolidated Framework for Implementation Research (CFIR) interview questions involving ED staff, leadership, and outpatient community partners familiar with the program [66]. Individuals cannot participate in the qualitative interviews if they are unfamiliar with the ED-initiated buprenorphine program. Additionally, adoption includes the total number of ED staff trained in the ED-initiated buprenorphine protocol. Maintenance/Sustainability will be maintained through forming a long-term sustainability plan and obtaining continued funding if the program is successful.

Furthermore, implementation will be measured by creation of a new clinical workflow as well as self-reported patient outcomes. Any rural hospital ED patient who receives buprenorphine, speaks English, and can provide follow-up contact information may complete post-discharge surveys to further assess implementation outcomes. Surveys include the Patient Satisfaction Questionnaire Short Form (PSQ-18), to assess their ED experience with buprenorphine initiation, [67] and the Center for Substance Abuse Treatment (CSAT) Government Performance and Results Act (GPRA) Client Outcome Measures for Discretionary Programs [68]. Grant funding requirements included CSAT GPRA survey collection for outcome measurements at a federal level [69]. CSAT GPRA survey assesses 30-day substance use diagnosis and treatment, social connectedness, living conditions, mental and physical health problems, legal status, education, employment, and income. Patients are contacted by the patient navigator or a research assistant within 1 business day from being seen in the ED to complete the PSQ-18 and CSAT GPRA. Patients are also contacted 6-months after their index ED visit by a research assistant to complete a follow-up GPRA survey.

The community hospital coordinators will collect and record data from the EMR into aggregate case logs. The implementation team monitors case logs weekly to adapt intervention delivery as needed. Collaborating external facilitators will conduct anonymous semi-structured qualitative interviews after obtaining informed consent. The qualitative interviews will be transcribed and coded by a study coordinator and staff with prior qualitative experience as a part of research. Anonymous patient GPRA and PSQ-18 survey informed consent will be obtained by a research coordinator or the patient navigator. Participants receive $30 gift cards upon completion of each qualitative interview and survey. Findings will be shared during weekly and monthly implementation stakeholder meetings. De-identified aggregate data will be disseminated as national poster presentations and journal articles. All data is stored in password protected electronic files. All data will be destroyed upon study completion.

**Table 3:** Primary outcome measure summary

Measure	Description
OUD Screening	• The number of ED patients screened for OUD with the DSM-5.
Buprenorphine Initiation	• The number of ED patients initiated on buprenorphine in the ED or discharged with a buprenorphine prescription and home initiation instructions.
MOUD Follow Up	• The number of patients who attended their initial outpatient clinician appointment for ongoing MOUD and subsequent weekly appointment attendance.
Naloxone Distribution	• The number of ED patients who received naloxone rescue kits.
Overdose Reversal Training	• The number of ED patients trained in naloxone administration by ED staff.

## Discussion and conclusion

Rural communities continue to face significant morbidity and mortality from the opioid epidemic. ED-initiated buprenorphine is an opportune setting to address OUD, especially in rural locations facing additional treatment barriers. Though ED-initiated buprenorphine has been implemented across multiple settings, few studies have assessed rural implementation and no known studies have been done in the rural Mountain West.

Multiple rural specific implementation barriers exist. However, external facilitation has been successfully incorporated in prior ED-initiated buprenorphine studies and has the potential to address these barriers. Combining the expertise from an academic institution with local community partners could help identify valuable implementation strategies for effective rural ED-initiated buprenorphine program implementation. Further disseminations of successful implementation strategies offering more accessible OUD interventions can help address rural disparities and prevent subsequent morbidity and mortality.

## Data Availability

Not applicable.

## Abbreviations

COVID-19: Coronavirus disease of 2019
ED: Emergency department
OUD: Opioid use disorder
SUD: Substance use disorder
MOUD: Medication for opioid use disorder
DATA: Drug addiction treatment act
RE-AIM: Reach, effectiveness, adoption, implementation, and maintenance
DSM-5: Diagnostic and statistical manual of mental disorders fifth edition
MFM: Maternal fetal medicine
ACEP: American college of emergency physicians
DEA: Drug enforcement agency
EMR: Electronic medical record
COWS: Clinical opioid withdrawal scale
CSA: Controlled substances act
APC: Advanced practice clinician
CSAT GPRA: Center for Substance Abuse Treatment Government Performance and Results Act
